# Pro-inflammatory cytokines in aqueous humor from dogs with anterior uveitis and post-operative ocular hypertension following phacoemulsification, primary glaucoma, and normal healthy eyes

**DOI:** 10.1371/journal.pone.0273449

**Published:** 2022-08-23

**Authors:** Hannah M. Terhaar, Michala de Linde Henriksen, Lisa K. Uhl, Corey Boeckling, Carolina Mehaffy, Ann Hess, Michael R. Lappin

**Affiliations:** 1 Department of Clinical Sciences, Comparative Ophthalmology, College of Veterinary Medicine and Biomedical Sciences, Colorado State University, Fort Collins, CO, United States of America; 2 Pathology, Department of Veterinary Pathology, College of Veterinary Medicine, Iowa State University, Ames, IA, United States of America; 3 Bioanalysis and Omics (ARC-BIO), Colorado State University, Fort Collins, CO, United States of America; 4 Department of Microbiology, Immunology and Pathology, College of Veterinary Medicine and Biomedical Sciences, Colorado State University, Fort Collins, CO, United States of America; 5 Department of Statistics, College of Natural Sciences, Colorado State University, Fort Collins, CO, United States of America; 6 Department of Clinical Sciences, Center for Companion Animal Studies, College of Veterinary Medicine and Biomedical Sciences, Colorado State University, Fort Collins, CO, United States of America; SRM Institute of Science and Technology, INDIA

## Abstract

**Background:**

The aim of this study was to evaluate the levels of pro-inflammatory cytokines in aqueous humor (AH) from dogs with anterior uveitis and post-operative ocular hypertension (POH) following phacoemulsification, in AH from dogs with primary glaucoma, and in normal healthy eyes with no signs of anterior uveitis or other ocular diseases.

**Methods:**

An exploratory study including 21 samples of AH collected from 15 dogs; post-phacoemulsification with anterior uveitis and POH (‘POH group’, n = 10 samples), primary glaucoma (‘glaucoma group’, n = 6 samples), and normal (‘normal group’, n = 5 samples). Target mass spectrometry via multiple reaction monitoring (MRM-MS) with the Canine Cytokine SpikeMix™ as internal standard was used to measure the pro-inflammatory cytokine levels.

**Results:**

The MRM-MS method measured 15 pro-inflammatory cytokines. Tumor-necrosis-factor-alpha (TNFα) and interleukin-18 (IL-18) levels in AH were different between all three groups (glaucoma>POH>normal) (*p =* .05, *p =* .02, respectively). Additionally, IL-6 was higher in the ‘POH group’ compared to the ‘glaucoma group’ (*p =* .04) and IL-4 was higher in the ‘POH group’ compared to the ‘normal group’ (*p =* .04). Intraocular pressure (IOP) was positively associated with increased AH levels of IL-18 (Spearman correlation = .64, *p* = .03).

**Conclusions:**

MRM-MS using the Canine Cytokine SpikeMix™ as an internal standard was established as a method to detect pro-inflammatory cytokine levels in canine AH. The study demonstrated increased levels of IL-4, IL-6, IL-18, and TNFα in AH from canines with POH following phacoemulsification. Primary glaucomatous eyes had the highest levels of IL-18 and TNFα which may indicate that inflammation plays a role in the pathogenesis of primary glaucoma in dogs.

## Introduction

Anterior uveitis is a painful and potentially blinding disease in humans and animals due to its ability to cause complications such as secondary glaucoma [1,2]. Breakdown of the blood aqueous barrier (BAB) by either exogenous or endogenous factors will cause anterior uveitis [3]. Exogenous factors include corneal ulcerations, cataracts causing lens induced uveitis, or trauma to the uvea from intraocular procedures such as cataract surgery (phacoemulsification) [3,4]. Endogenous factors, also known as ocular manifestations of systemic disease, include systemic infections, systemic neoplasia, or immune mediated disorders [3,4].

The two most common causes of cataracts in dogs are hereditary and diabetes mellitus [5]. Phacoemulsification causes significant anterior uveitis in the initial weeks following phacoemulsification (post-phaco). Anterior uveitis post-phaco is especially significant for dogs and human pediatric patients [6,7]. Significant post-phaco anterior uveitis, along with other contributing factors, can cause an elevation in intraocular pressure (IOP) which is distinct from glaucoma and referred to as post-operative ocular hypertension (POH) [6,8,9]. The time frames for POH reported in the literature vary, but it is described as a transient elevation in IOP within the first 24 hours through 14 days after surgery [6,10]. Retention of viscoelastic material in the anterior chamber has been implicated to increase the incidence of POH [11]. POH can lead to irreversible blindness due to retinal and optic nerve damage from the elevated IOP and there is a risk of chronic glaucoma; therefore, control of POH is an important factor in post-phaco patients [8].

Post-phaco anterior uveitis treatment for canine patients aims to control arachidonic acid-mediated inflammation by treatment with topical and systemic glucocorticoids and/or non-steroidal anti-inflammatory drugs (NSAIDs) [12]. These approaches have potential side effects, including gastrointestinal ulcers, renal failure, uncontrolled diabetes, and increased risk of corneal infections [13,14]. Finding alternative anti-inflammatory and immunotherapy treatment options has gained increased attention in ophthalmology research [15–18]. This can be facilitated by determining which pro-inflammatory cytokines are involved in post-phaco anterior uveitis. In humans with anterior uveitis, specific pro-inflammatory cytokines have been detected in aqueous humor (AH) including Interleukin (IL)-6, IL-8, IL-10, IL-13, IL-17, interferon-gamma (IFNγ), and tumor necrosis factor-alpha (TNFα) [19–22]. In dogs, IL-1β levels evaluated by enzyme-linked immunosorbent assay (ELISA) were increased in AH of eyes with cataracts [23]. In horses, IL-10, IL-17, IL-23, and IFNγ have shown potential for being important pro-inflammatory cytokines in horses with equine recurrent uveitis [24].

Various methods for measurement of pro-inflammatory cytokines in AH samples include polymerase chain reaction (PCR) assay, immunoassays such as ELISA, multiplexed or bead-based immunoassays, and flow cytometric analysis, as well as target mass spectrometry [19,22,25,26]. Multiplex bead assays have gained popularity due to their ability to permit examination of multiple analytes from a single sample, which is more rapid than other methods such as PCR and standard ELISA, however quality control and interlaboratory variation present challenges [27,28]. Additionally, multiplex assays can be impacted by unpredictable potential biological interactions between multiple cytokines and other molecules in a sample [29]. Target mass spectrometry may provide a more reliable and specific method of cytokine detection by evaluating proteins (cytokines) after enzymatic digestion and detection of selected precursor and transition ions from specific peptides [30]. Target mass spectrometry with the multiple reaction method (MRM-MS) has high specificity, high sensitivity, and multiplexing ability and appears to be a suitable alternative to immunoassays to screen for multiple cytokines. Additionally, MRM-MS allows the utilization of a single, small sample volume to quantify multiple low abundance analytes. This is beneficial in the evaluation of AH, because only limited fluid volume can be obtained from humans or small animals such as dogs [31]. Additionally, Canine Cytokine SpikeMix™ (JPT Peptide Technologies, Berlin, Germany) is a mixture of canine specific stable isotope labeled peptides that provides relative quantification of cytokines by mass spectrometry. Targeted proteomics with labeled peptides (by JPT peptide technologies) has been used in many different areas of research including biomarkers or diagnosis of osteoarthritis, Alzheimer’s disease including tear analysis, cancer and infectious diseases in humans [32–36]. Analysis with MRM-MS and Canine Cytokine SpikeMix™ could be a useful tool to characterize AH pro-inflammatory cytokines and monitor response to therapy in canine anterior uveitis.

The purpose of this study was to evaluate the levels of pro-inflammatory cytokines in AH from dogs with anterior uveitis and POH post-phaco, dogs with primary glaucoma, and in normal healthy eyes with no signs of anterior uveitis or other ocular diseases. MRM-MS was used as the method to evaluate the level of pro-inflammatory cytokines in AH. To the authors’ knowledge, this is the first study to describe pro-inflammatory cytokine levels in AH from dogs with anterior uveitis and POH post-phaco and primary glaucoma dogs. Additionally, this is the first study to evaluate the Canine Cytokine SpikeMix™ used as internal control in MRM-MS for its ability to provide relative quantification of pro-inflammatory cytokines in canine AH.

## Materials and methods

### Ethics statement

Owners of dogs admitted to Colorado State University Veterinary Teaching Hospital have signed a client consent form that gives permission to use information regarding their dog’s diseases in future studies. The aqueous humor samples that were collected from patients in this study were either collected as a part of their treatment for anterior uveitis and POH or following enucleation of the eye. Aqueous humor from healthy research Beagles were collected with permission from institutional animal care and use committee (IACUC, protocol #18-1234A).

### Study design and population

This is an exploratory study. Three groups were included in this study: The post-phacoemulsification group includes dogs with anterior uveitis and POH following surgery (‘POH group’). Primary glaucoma dogs (‘glaucoma group’). This group includes dogs with elevated intraocular pressure (IOP) due to primary / hereditary glaucoma from either goniodysgenesis or anterior segment dysgenesis. The normal control dogs (‘normal group’). This group includes healthy research Beagle dogs with no signs of anterior uveitis or other ocular diseases. The ‘POH group’ and ‘glaucoma group’ were clinical patients at Colorado State University Veterinary Teaching Hospital between 2018–2019.

### Ophthalmic examination

All dogs in this study received a physical examination by an ophthalmology resident and a complete ophthalmic examination by a board-certified veterinary ophthalmologist (DACVO) and by an ophthalmology resident at every appointment/rechecks and prior to any procedures such as phacoemulsification, aqueous paracentesis, or enucleation. A complete ophthalmic examination included the following steps: neuro-ophthalmic examination included menace response, palpebral reflex, dazzle reflex, and indirect and direct pupillary light reflex. The adnexa and anterior segment were examined with slit-lamp biomicroscopy (SL-17; Kowa Company Ltd., Tokyo, Japan), and the posterior segment was examined using an indirect ophthalmoscopy headset (Keeler Vantage; Keeler Instruments, Inc., Broomall, PA, USA) and a 28 diopter condensing lens (Volk; Mentor, OH, USA). Dilated indirect ophthalmoscopy was performed on the ‘normal group’ but not on the ‘POH group’ or ‘glaucoma group’ due to contraindication for dilation with elevated IOP [37–40]. Dilation of the pupil was facilitated by instillation of one drop (0.05 ml) tropicamide 1% ophthalmic solution (Akorn, Lake Forest, IL, USA) to both eyes. Diagnostic tests including tear production measurement with Schirmer tear test (Eye Care Product Manufacturing, LLC, Tuscon, AZ, USA), IOP measurement with rebound tonometry (Tono-Vet; Icare® Finland Oy, Espoo, Finland), and fluorescein stain to evaluate for corneal ulceration (Jorgensen Lab, Loveland, CO) were performed on all eyes.

### The ‘POH group’

The ‘POH group’ consisted of dogs that underwent phacoemulsification for diabetic or inherited cataracts and developed anterior uveitis and POH within the first seven days post-phaco (Table 1). Inclusion criteria consisted of IOP elevation of ≥ 25 mmHg within the first seven days following phacoemulsification and with clinical signs of anterior uveitis including aqueous flare subjectively graded by the Kimura flare scale 0 to 4+ [41] by a DACVO and an ophthalmology resident. AH was collected from these dogs as a part of their treatment for anterior uveitis and POH post-phaco (see protocol below).

**Table 1:** Clinical information of all patients.

Group	ID number	Patient information	Eye	Time of collection	Ophthalmic disease	IOP^1^ (mmHg)	IOP^2^ (mmHg)	Flare
POH	01	9 year old FS Labrador Retriever	OD	1 day post-phacoemulsification	Diabetic cataracts	12	45	3+
			OS	1 day post-phacoemulsification	Diabetic cataracts	50	50	4+
			OS	6 days post-phacoemulsification	Diabetic cataracts	25	50	2+
POH	02	10 year old FS English Springer Spaniel	OD	1 day post-phacoemulsification	Diabetic cataracts	29	48	1+
			OS	1 day post-phacoemulsification	Diabetic cataracts	35	40	1+
POH	03	10 year old MC Labrador Retriever	OS	1 day post-phacoemulsification	Diabetic cataracts	48	48	3+
			OD	5 days post-phacoemulsification	Diabetic cataracts	31	31	3+
POH	04	8.5 year old MC Welsh Terrier	OD	2 days post-phacoemulsification	Diabetic cataracts	12	33	3+
			OS	2 days post-phacoemulsification	Diabetic cataracts	26	26	3+
POH	05	11 year old FS Cocker Spaniel	OD	1 day post-phacoemulsification	Hereditary cataract (hypermature)	23	50	3+
Glaucoma	06	2 year old FS Newfoundland	OD	Post-enucleation	Glaucoma, anterior segment dysgenesis*	65	65	3+
Glaucoma	07	2.5 year old FS mixed breed	OS	Post-enucleation	Glaucoma, goniodysgenesis*	6	72	2+
Glaucoma	08	12 year old MC mixed breed	OS	Post-enucleation	Glaucoma, goniodysgenesis*	69	74	1+
Glaucoma	09	5 year old MC English Springer Spaniel	OD	Post-enucleation	Glaucoma, goniodysgenesis*	23	85	Trace
Glaucoma	10	10.5 year old MC West Highland White Terrier	OD	8 days post-phacoemulsification	Glaucoma, goniodysgenesis†	60	60	Trace
			OS	8 days post-phacoemulsification	Glaucoma, goniodysgenesis†	54	54	Trace
Normal	11	1.5 year old MI Beagle	OS	Aqueous paracentesis	Normal	19	NR	0
Normal	12	3 year old MI Beagle	OD	Aqueous paracentesis	Normal	19	NR	0
Normal	13	3.5 year old MI Beagle	OD	Aqueous paracentesis	Normal	18	NR	0
Normal	14	2.5 year old FI Beagle	OS	Aqueous paracentesis	Normal	18	NR	0
Normal	15	1.5 year old FI Beagle	OD	Aqueous paracentesis	Normal	20	NR	0

Aqueous humor (AH) samples were collected from three groups: ‘POH group’ (n = 10): Dogs with anterior uveitis and post-operative ocular hypertension (POH) following phacoemulsification, ‘Glaucoma group’ (n = 6): Dogs with primary glaucoma, ‘Normal group’ (n = 5): Healthy research beagles with no sign of anterior uveitis or other ocular diseases. Patient information, eye (OD = right eye, OS = left eye), time of collection, ophthalmic disease, intraocular pressure (IOP)^1^ –the day of AH collection, IOP^2^ –highest IOP measured for this patient, and subjective assessment of aqueous flare are described.

*confirmed via histopathology

†confirmed with gonioscopy, NR = not recorded. FI = female intact; FS = female spayed; MI = male intact; MC = male castrated.

#### Perioperative phacoemulsification protocol

All dogs in the ‘POH group’ were treated with variable combinations of the follow medications prior to surgery (Table 2A): ofloxacin 0.3% ophthalmic solution (Apexa, Boise, ID), diclofenac 0.1% ophthalmic solution (Sandoz, Princeton, NJ), ketorolac 0.5% ophthalmic solution (Sandoz, Princeton, NJ), prednisolone acetate 1% ophthalmic suspension (Sandoz, Princeton, NJ), I-drop® Vet Plus ophthalmic lubrication (I-MED Animal Health, Dollard-des-Ormeaux, QC, Canada), OptixCare ophthalmic lubrication (Aventix, Burlington, Ontario, Canada), dorzolamide 2% ophthalmic solution (Bausch & Lomb, Bridgewater, NJ), and dorzolamide 2%/timolol 0.5% ophthalmic solution (Cosopt, Sandoz, Princeton, NJ). Treatment with diclofenac versus ketorolac was based on pre-referral medications started by the primary veterinarian. If the patient was not on a topical NSAID at the time of referral, they were placed on diclofenac 0.1% ophthalmic solution. Treatment with dorzolamide 2% versus dorzolamide 2%/timolol 0.5% was based on clinician preference and cardiovascular health of the patient. Timolol is a beta-blocker and results in a decreased heart rate when applied topically in canines [42]. On the morning of surgery, presurgical treatment consisted of the following topical drugs given in alternation every five minutes for two to three doses per drug: tropicamide 1% ophthalmic solution, phenylephrine 2.5% ophthalmic solution (Paragon BioTeck, Inc., Portland, OR), diclofenac 0.1% ophthalmic solution, prednisolone acetate 1% ophthalmic suspension, and ofloxacin 0.3% ophthalmic solution.

**Table 2:** Pre- and post-operative surgical information for the ‘POH group’ and ‘glaucoma group’.

A	ID number	Eye	Oral Medications	Ketorolac or Diclofenac	Ofloxacin	Dorzolamide or Dorzolamide/Timolol	Prednisolone	Phacoemulsification time (seconds)	IOL placed
**POH group (pre-sx)**	01	OD	none	+	+	+	+	4:30.70	+ 14mm
		OS						6:42.20	+ 14mm
	02	OD	none	+	-	-	+	NR	+ 13mm
		OS						NR	+ 13mm
	03	OS	none	+	-	-	-	NR	+ 14 mm
		OS						NR	-
	04	OD	Carprofen	+	-	+	+	2:48.30	-
		OS						4:17.00	-
	05	OD	none	+	-	+	-	NR	+ 13 mm
**B**	**ID number**	**Eye**	**Oral Medications**	**Ketorolac or Diclofenac**	**Ofloxacin**	**Dorzolamide or Dorzolamide/Timolol**	**Latanoprost**	**Prednisolone**	**Eye lubricant**
**POH group (post-sx)**	01	OD	Carprofen, Clavamox	+	+	+	+	+	+
		OS							
	02	OD	Carprofen, Clavamox	+	+	+	+	+	+
		OS							
	03	OS	Carprofen, Clavamox	+	+	+	+	+	+
		OS				-	-		
	04	OD	Carprofen, Clavamox	+	+	+	+	+	+
		OS							
	05	OD	Prednisone, Cefpodoxime	+	+	+	+	+	+
**C**	**ID number**	**Eye**	**Dorzolamide or Dorzolamide/Timolol**	**Latanoprost**	**Prednisolone**	**Other**		**Phacoemulsification time (seconds)**	**IOL placed**
**Glaucoma group**	06	OD	+	+	+	-		NA	NA
	07	OS	+	+	-	Eye lubricant		NA	NA
	08	OS	+	+	+	-		NA	NA
	09	OD	+	-	+	-		NA	NA
	10	OD	+	-	+	Diclofenac, Ofloxacin, Cefpodoxime PO		NR	+ 13 mm
		OS						NR	+ 13 mm

A) Pre-surgical and surgical characteristics (pre-sx) from the ‘POH group’ including pre-operative medications, phacoemulsification times (UST) when available, and if an intraocular lens (IOL) was placed (+ = IOL placed;— = no IOL placed). B) Post-surgical (post-sx) medications administered to the ‘POH group’. C) Medications prior to enucleation or aqueous paracentesis for the ‘glaucoma group’. Phacoemulsification times and if an IOL was placed are reported for one patient (patient 10) that developed glaucoma > 7 days post-phacoemulsification with no evidence of anterior uveitis. (+ = this medication(s) was used on this patient,— = this medication(s) was not used on this patient, NA = not applicable, NR = values not recorded in medical record).

Between anesthetic induction and beginning surgery, cefazolin (Hospira Inc., Lake Forest, IL) was administered intravenously (22 mg/kg); this was repeated every 90 minutes during surgery. A routine one‐handed phacoemulsification procedure was performed by a DACVO or by an ophthalmology resident under direct supervision of a DACVO. Following a 3‐mm clear corneal incision performed 1mm from the limbus, Trypan blue 0.3 mL (I‐Med Animal Health, Dollard‐des‐Ormeaux, QC, Canada) was injected into the anterior chamber to visualize the anterior lens capsule and facilitate capsulorrhexis. Trypan blue was followed by viscoelastic (I‐Visc 1.8%, i‐Medical® Ophthalmic International Heidelberg GmbH, Mannheim, Germany) injected into the anterior chamber to retain its form and protect corneal endothelial cells from trauma and heat caused by the phacoemulsification process. The capsulorrhexis was performed by using a 22‐gauge needle to create a hole in the anterior lens capsule. Utrata capsulorrhexis forceps were used to perform an approximately 4‐5 mm diameter continuous curvilinear capsulorrhexis, after which phacoemulsification of the lens was performed. The AMO Whitestar Signature phacoemulsification instrument (Johnson and Johnson Surgical Vision, Inc.; Santa Ana, CA) was used for all phacoemulsification surgeries. Irrigation/aspiration was used to remove any remaining cortex followed by placement of intraocular lenses (IOLs) (Acrivet, Bausch & Lomb, Bridgewater, NJ) in eyes where no contraindications such as posterior lens capsule rupture or tear were identified. Irrigation and aspiration of the anterior chamber to remove viscoelastic device were then performed for at least one minute in all eyes. The corneal incision was closed with three to four simple interrupted sutures using 8‐0 Vicryl suture (Ethi-con, Somerville, NJ). Phacoemulsification times as well as IOL placement is documented for cases when available in Table 2.

Sequential tonometry was performed one hour after the dog was extubated and every one to two hours thereafter until the dog was discharged approximately five to six hours after surgery. Monitoring IOPs following phacoemulsification is common practice in veterinary ophthalmology for monitoring and early intervention of POH [6,43,44]. Topical medications were continued for three to four weeks postoperatively and are recorded in Table 2B. Patients were discharged on an oral antibiotic as well as an oral anti‐inflammatory (NSAID or steroid) for three to four weeks postoperatively (Table 2). All dogs were discharged the day of surgery and returned for recheck examination one day postoperatively as well as five to seven days postoperatively.

#### Aqueous paracentesis and tissue plasminogen activator injection

Dogs with IOPs ≥ 25 mmHg within the first seven days of surgery had an aqueous paracentesis performed for clinical management. The aqueous paracentesis allowed 0.2 ml of AH to be removed from the anterior chamber, followed by injection of 25 micrograms tissue plasminogen activator (TPA, Activase®, Genentech, San Francisco, CA). The aqueous paracentesis and TPA injection were performed to decrease IOP and to clear the iridocorneal angle of potential inflammatory debris such as fibrin.

Dogs were sedated based on clinician assessment of the patient temperament for an aqueous paracentesis to be safely performed. Prior to aqueous paracentesis, eyes were flushed three times with betadine solution (diluted 1:50) followed by application of three rounds (0.1 ml per round) of topical analgesia with proparacaine 0.5% ophthalmic solution (Sandoz, Princeton, NJ) applied to the eye. All eyes had a 27-gauge needle connected to a 1.0-milliliter syringe placed into the anterior chamber at the dorsal limbus by a veterinary ophthalmology resident supervised by a DACVO. Care was taken to avoid the corneal endothelium, iris, and lens. Following insertion into the anterior chamber, 0.2ml of AH was collected. The 1.0-milliliter syringe was removed and a new 1.0-milliliter syringe containing 25 micrograms TPA was connected to the 27-guage needle for injection into the anterior chamber. The needle with syringe was removed from the anterior chamber and the insertion site at the limbus was compressed with a 0.3mm Bishop Harmon forceps for 30 seconds to prevent leakage of AH. The AH samples were stored in a -80C freezer within 10 minutes of the aqueous paracentesis until analysis. Following aqueous paracentesis and TPA injection, the IOP was measured to verify the IOP was below 25 mmHg.

### The ‘glaucoma group’

The ‘glaucoma group’ consisted of dogs with an elevated IOP and minimal to no evidence of clinical anterior uveitis. Inclusion criteria consisted of an elevated IOP > 25 mmHg, evidence of blindness on neuro-ophthalmic examination (negative menace, dazzle, and PLR) and diagnosed with primary / hereditary glaucoma confirmed as goniodysgenesis or anterior segment dysgenesis by histopathologic analysis of an enucleated eye or gonioscopy performed by a DACVO (Table 1). Table 2C documents the anti-glaucoma drugs and other topical medications dogs had received prior to AH collection. When eyes from the ‘glaucoma group’ were enucleated, a routine subconjunctival enucleation was performed [45], and AH (0.2 mL) was collected with an aqueous paracentesis immediately following removal of the globe from the surgical field with a 27g needle and 1.0-mililiter syringe. The AH samples were stored in a -80C freezer within 10 minutes after the aqueous paracentesis until analysis. The enucleated globes were placed in 10% neutral buffered formalin (Cole-Parmer, Vernon Hills, IL) and submitted to the Comparative Ocular Pathology Laboratory of Wisconsin (COPLOW; Madison, WI) for histopathology evaluation by board-certified veterinary pathologists.

### The ‘normal group’

Dogs in the ‘normal group’ consisted of healthy research Beagles with normal IOP values and no signs of anterior uveitis or other ocular diseases on complete ophthalmic examination performed by a DACVO (MdLH) and an ophthalmology resident (Table 1).

#### Aqueous paracentesis

Normal healthy dogs with a normal ophthalmic examination were sedated with 0.3–0.4 mg/kg of IV butorphanol tartrate; 15 minutes following injection, the aqueous paracentesis procedure was performed. The aqueous paracentesis was performed as described above but with no TPA injection. Instead, the 27-gauge needle with syringe was removed from the anterior chamber and the penetrated area at the limbus was compressed with a 0.3 mm Bishop Harmon forceps for 30 seconds to prevent leaking of AH. The 0.2 ml AH was immediately stored in separate Eppendorf tubes in a -80°C freezer until analysis. All eyes were administered one drop (0.05ml) of topical ofloxacin 0.3% ophthalmic solution, one drop (0.05ml) of prednisolone acetate 1% ophthalmic suspension, one drop (0.05 ml) of atropine 1% ophthalmic solution (Apexa, Boise, ID), and one drop (0.05 ml) of I-drop Vet eye lubrication administered immediately following the procedure, but with five minutes between each eye medication.

#### Post-aqueous paracentesis treatment for ‘normal group’

Aqueous paracentesis induces anterior uveitis via BAB breakdown [46,47], therefore the ‘normal group’ of dogs were treated with topical ophthalmic medications according to the following procedure: ofloxacin 0.3% ophthalmic solution one drop (0.05 ml) two times daily for five days and prednisolone acetate 1% ophthalmic suspension one drop (0.05 ml) two times daily for five days. Fluorescein stain was performed, and IOP measurement by tonometry was performed every day for five days post-procedure. Five days following the procedure, all dogs had a complete ophthalmic examination (as described under ‘ophthalmic examination’) performed to ensure all dogs had a normal ophthalmic examination with no signs of anterior uveitis before all topical medications were discontinued.

### AH analysis with MRM-MS

The AH samples were submitted for analysis of pro-inflammatory cytokines to Colorado State University’s Bioanalysis and Omics (ARC-BIO) facility.

#### Development of an MRM-MS method to detect inflammatory cytokines in canine AH: Sample processing, preparation, and analysis

AH samples were concentrated using a 3KDa Molecular-Weight-Cut-Off Spin column (Amicon). Concentrated proteins (retentate) were subjected to three washes (buffer exchanges) with 10mM ammonium bicarbonate and then quantified in the nanodrop. When possible, 50ug of each sample was subjected to trypsin digestion followed by clean up using a C18 column as previously described [48]. Briefly, samples were reduced and alkylated with 5mM dithiothreitol and 5mM iodoacetamide. Trypsin (Pierce MS-Grade, Thermo Scientific) was added at an enzyme to substrate ratio of 1:25 and incubated at 37°C for 3-hours. Trypsin was deactivated with the addition of 5% trifluoroacetic acid and desalted using Pierce C18 spin columns (Thermo Scientific) using the manufacturer’s instructions. Peptide eluate was dried in a vacuum evaporator. Digested samples were resuspended to an estimated final concentration of 2 μg/μl of the original protein concentration using 1:10 SpikeMix™-labeled peptides. Final peptide concentrations for each digested sample were estimated via nanodrop and final concentration was adjusted to 0.3μg/μl for all samples. A pool from all samples (1μl each) was made and used for optimization of the MRM-MS method.

An MRM-MS method to detect 15 pro-inflammatory cytokines; IL-1α, IL-1β, IL-3, IL-4, IL-5, IL-6, IL-8, IL-12α, IL-12β, IL-13, IL-18, TNFα, IFNγ, granulocyte-macrophage colony stimulating factor 2 (GMCSF2), and thrombopoietin (TPO) was developed using the Canine Cytokine SpikeMix^TM^ (JPT Peptide Technologies: SpikeMix™ Cytokines Canine–Heavy (Cat # SPT-CYT-POOL-L-can). This mix contains heavy labeled peptides corresponding to 145 peptides from 68 canine inflammatory proteins. Peptide Mix was resuspended in 1 ml of TQS loading buffer (3% ACN, 0.1% FA in water). Peptide sequences for the 15 targets was imported into the Skyline software [49]. MassLynx was used to operate the Xevo TQ-S mass spectrometer (Waters corp) coupled to a M-class UPLC (Waters corp) and Trizaic source. Peptides were separated in an ion key column (iKey, 150 μm x 50mm Peptide BEH C18, 13Å, 1.7 μm). The mix was used to identify the best performing transition ions and ideal chromatographic conditions. Once a preliminary method was established, a series of iterations (2 μl injections of the 1:10 SpikeMix™ dilution spiked into a pool of canine AH (made of 1ul of each sample)) were carried out to optimize the method by identifying the best collision energy for all targets. The final method was used to test each of the individual samples separately [49].

The final optimized MRM-MS method included two methods which contained 15 targeted proteins and 28 representative peptides. One contained 15 target peptides and the other contained 13. Each method was optimized so at least 12 points per peak were captured for each transition ion. In addition, a second round of methods, each containing three transitions per peptide (instead of the usual five) and only one peptide per protein target, was also used for analysis to increase the sensitivity of the methods with at least 20 points per peak captured. The final optimized LC gradient was as follows: 10 minutes linear gradient from 5% Buffer B (0.1% formic acid in Acetonitrile), 95% Buffer A (0.1% formic acid in water) to 45% buffer B, 55% Buffer A, followed by a wash at 95% Buffer B, 5% Buffer A for five minutes and final equilibration at 5% Buffer B, 95% Buffer A for five minutes. The total time for each method was 20 minutes.

Results were presented as normalized Total Peak Area (nTPA) as exported by Skyline. In brief, TPA (corresponding to all monitored transitions per peptide) was normalized against the TPA of the corresponding heavy monitored transitions (from the canine cytokine SpikeMix^TM^).

### Statistical analysis

IOP values (mean ± SD) were compared between all three groups using a one-way analysis of variance (ANOVA). To assess pairwise comparisons between IOP values for ‘POH’ and ‘glaucoma groups’, unpaired Student’s t-test was used.

Summary statistics (min, median, max) were calculated for pro-inflammatory cytokine levels in each group. Summary plots indicate that many of the cytokines were not normally distributed (including zero values and some outliers), hence non-parametric approaches were used. Kruskal-Wallis test was used to compare pro-inflammatory cytokine levels and IOP values between the three groups (‘POH group’, ‘glaucoma group’, ‘normal group’). Dwass, Steel, Critchlow-Fligner (DSCF) method was used for further pairwise comparisons between groups (‘glaucoma group’ versus ‘normal group’, ‘POH group’ versus ‘glaucoma group’, and ‘normal group’ versus ‘POH group’). To assess correlation between IOP and each cytokine, Spearman correlations were calculated. Raw *p-*values were calculated, and Bonferroni’s method was used to account for multiple testing (corresponding to multiple pro-inflammatory cytokines) when a raw *p*-value was *<* .05. Analysis was done in SAS 9.4 (SAS Institute Inc., Cary, NC).

## Results

### Samples collected

Twenty-one (21) samples of AH from 15 dogs were collected and submitted for processing and analysis with MRM-MS (Table 1). The ‘POH group’ had 10 AH samples collected from five dogs (nine eyes) with one eye collected at two different time points. Dogs ranged in age from 1.5–11 years: one dog had a unilateral hereditary cataract (due to enucleation of the other globe), the remaining four dogs had bilateral diabetic cataracts. The ‘glaucoma group’ had six AH samples collected from five dogs (six eyes) ranging in age from 2–12 years of age: four samples were post-enucleation and histopathology revealed goniodysgenesis in three of the enucleated eyes and one eye was diagnosed with anterior segment dysgenesis. Other histopathology findings were similar among all eyes including optic nerve head cupping, gliosis, retinal atrophy, and minimal inflammation. Two samples in the ‘glaucoma group’ were from a single dog (via aqueous paracentesis) that developed glaucoma and blindness eight days post-phaco. This dog was diagnosed with goniodysgenesis pre-phacoemulsification on its gonioscopy examination. Minimal signs of bilateral anterior uveitis (trace flare) were found on this dog’s ophthalmic examination prior to aqueous paracentesis. Aqueous paracentesis and TPA injection were performed as a last resort to decrease IOPs but with no effect. Due to the absence of visible evidence of anterior uveitis, the diagnosis of goniodysgenesis, and elevated IOPs with no effect of TPA, this dog was placed in the ‘glaucoma group’. Euthanasia was elected by the owner due to bilateral glaucoma, but necropsy was declined so histopathology is not available. In the ‘normal group’, five AH samples were collected from five healthy research Beagles with no signs of anterior uveitis or other ocular pathology. One eye was randomly picked from each dog. The dogs age ranged from 1.5–3.5 years.

### MRM-MS testing of AH samples

The IOP prior to AH collection was highest in the ‘glaucoma group’ (46.2 ± 25.6 mmHg), followed by the ‘POH group’ (29.1 ± 12.8 mmHg) and the ‘normal group’ (18.8 ± 0.8 mmHg) with *p* = .03 (Table 1). With pairwise comparison between the ‘glaucoma group’ and ‘POH group’ immediately prior to AH collection, the ‘glaucoma group’ had the highest IOP followed by ‘POH group’, without statistical significance (*p* = .09). However, when comparing the highest recorded IOP for each patient, ‘glaucoma group’ (68.3 ± 11.0 mmHg) was higher than the ‘POH group’ (42.1 ± 9.0 mmHg) (*p* < .01).

**Fig 1:**
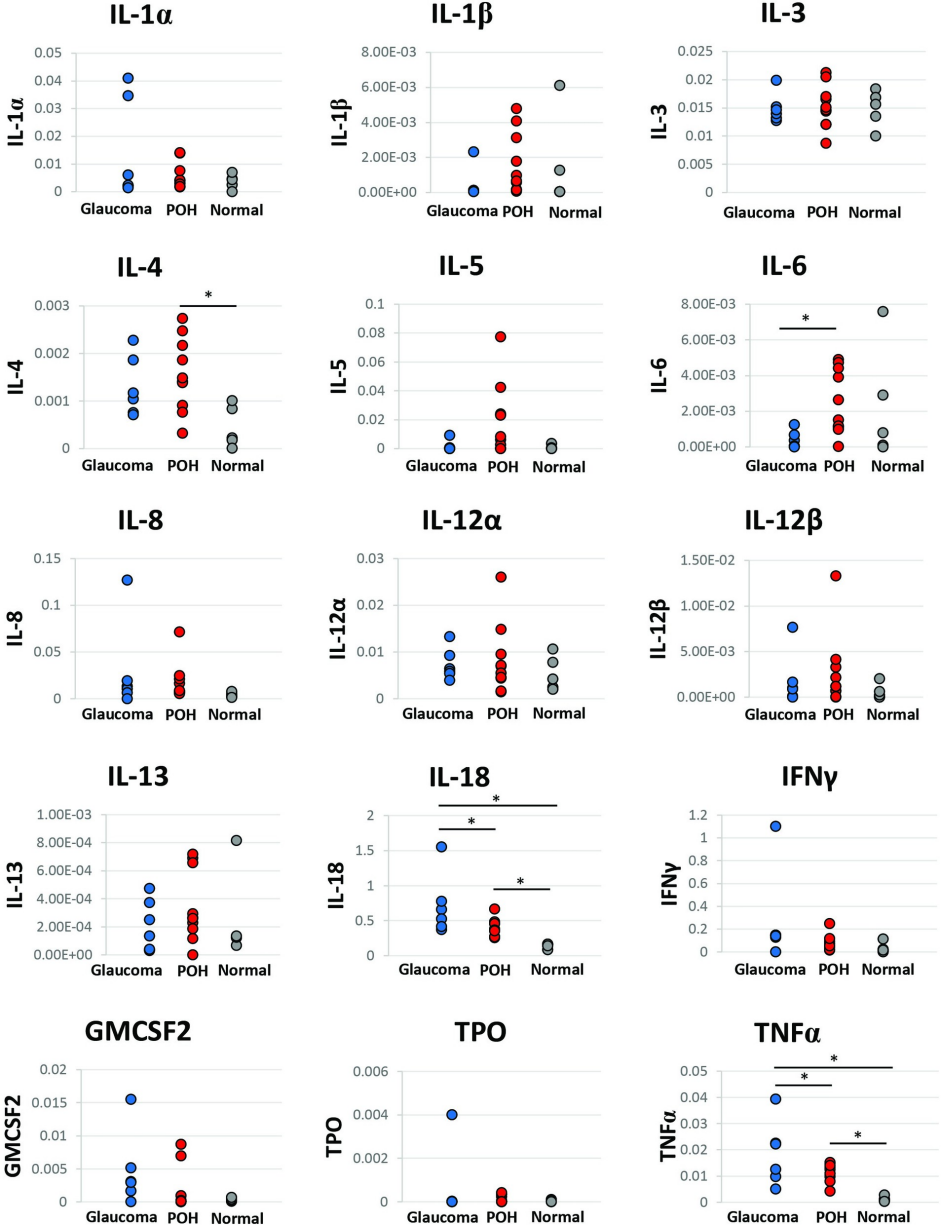
Cytokine levels in canine aqueous humor. Cytokine levels (normalized Total Peak Area, nTPA) in the aqueous humor (AH) of canines including Interleukin (IL) 1α, IL-1β, IL-3, IL-4, IL-5, IL-6, IL-8, IL-12α, IL-12β, IL-13, IL-18, Tumor necrosis factor-alpha (TNF-α), Interferon-gamma (IFNγ), granulocyte-macrophage colony stimulating factor 2 (GMCSF2), and thrombopoietin (TPO). TNFα and IL-18 levels in AH were different between all three groups with the ‘glaucoma group’ having the highest levels, followed by the ‘POH group’, then the ‘normal group’ (*p* = .05 and *p =* .02, respectively). The ‘POH group’ (eyes with anterior uveitis and post-operative ocular hypertension following phacoemulsification) had higher levels of IL-6 and IL-4 when compared to the ‘glaucoma group’ and ‘normal group’, respectively. (*p* = .04 and *p* = .04, respectively). *P* ≤ .05 is indicated with a solid black line and an asterisk.

**Fig 2:**
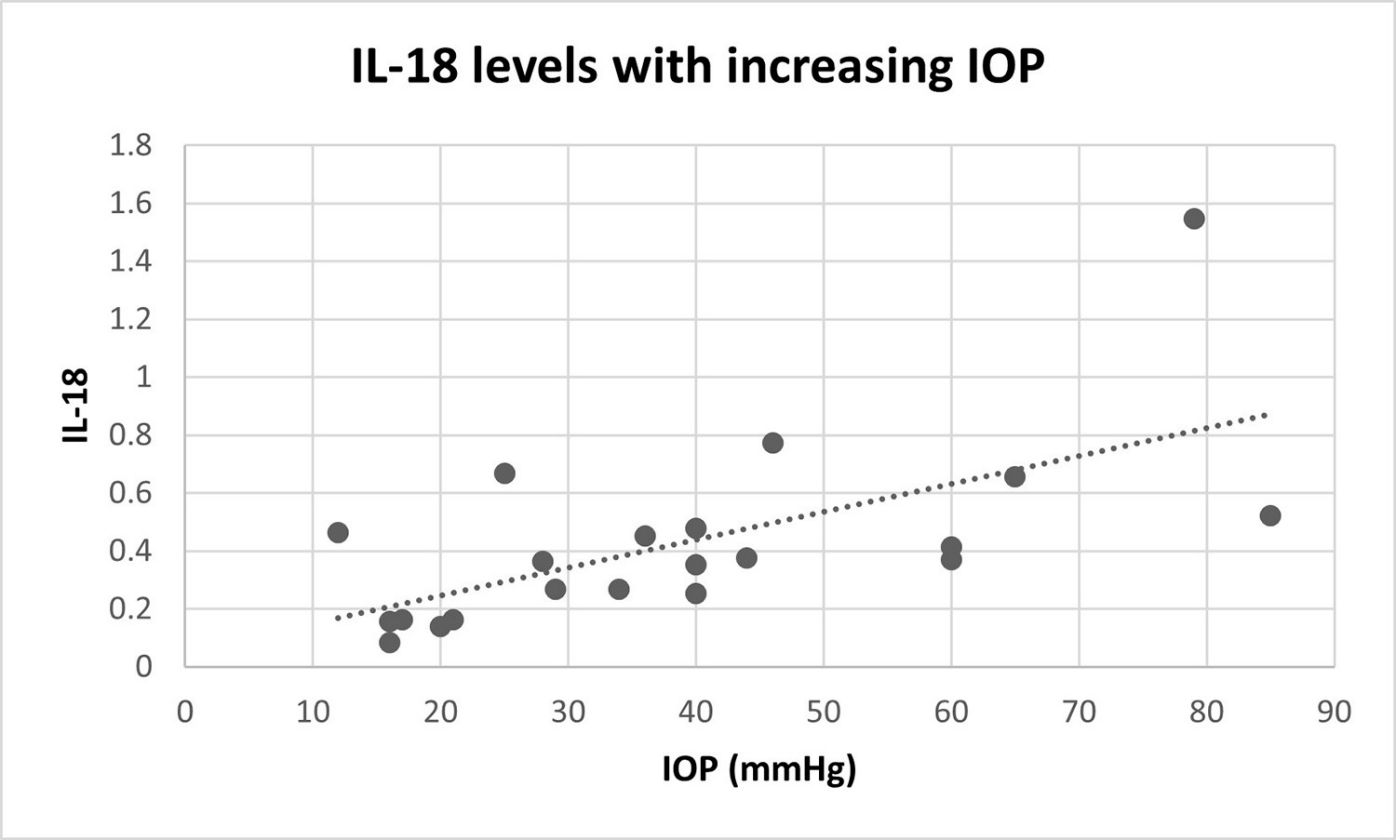
IL-18 and intraocular pressure. IL-18 levels (normalized Total Peak Area, nTPA) in the aqueous humor of canines were positively correlated with intraocular pressure (Spearman correlation = .64, *p* = .03).

Table 3 summarizes the pro-inflammatory cytokine levels for each group. TNFα and IL-18 levels in AH were different among all three groups with the ‘glaucoma group’ having the highest levels followed by the ‘POH group’ and ‘normal group’ (TNFα *p =* .05 and IL-18 *p =* .02). When comparing AH levels of IL-6, the ‘POH group’ had higher levels compared to the ‘glaucoma group’ (*p* = .04), but there was no evidence of differences between the ‘glaucoma group’ and ‘normal group’ (*p* = .57) or the ‘normal group’ and ‘POH group’ (*p* = .67). For IL-4, the ‘POH group’ had higher levels compared to the ‘normal group’ (*p* = .04). There was no evidence of differences between the ‘glaucoma group’ and ‘normal group’ (*p* = .11) or the ‘glaucoma group’ and ‘POH group’ (*p* = .72). There was no evidence of differences between the groups in pro-inflammatory cytokine levels for IL-1α, IL-1β, IL-3, IL-5, IL-8, IL-12α, IL-12β, IL-13, IFNγ, GMCSF2, and TPO (Fig 1). The AH levels of IL-18 were positively correlated with increasing IOP (Spearman correlation = .64, *p* = .03) (Fig 2).

**Table 3:** Pro-inflammatory cytokine levels for all groups.

Cytokine	Glaucoma	POH	Normal	Raw *P*-value	Bonferroni *P*-value
IL-1α	41 (13, 410)	36 (17, 140)	42 (0, 69)	0.92	
IL-1β	1.0 (0.47, 23)	8.2 (0.69, 48)	0.35 (0.30, 61)	0.12	
IL-3	140 (120, 200)	150 (87, 210)	160 (100, 180)	0.95	
IL-4	11 (7.1, 23)	14 (3.2, 27)	2.2 (0.052, 10)	0.031*	0.49
IL-5	2.1 (0, 35)	71 (0, 0770)	2.1 (0, 35)	0.13	
IL-6	2.0 (0, 12)	21 (0.091, 49)	7.9 (0, 76)	0.065	
IL-8	120 (0, 1300)	81 (53, 700)	35 (11, 74)	0.037*	0.60
IL-12α	61 (39, 130)	62 (14, 260)	42 (20, 100)	0.74	
1L-12β	9.4 (0.12, 76)	12 (0.18, 130)	2.3 (0, 20)	0.21	
IL-13	1.9 (0.29, 4.7)	2.6 (0, 7.2)	1.2 (0, 82)	0.53	
IL-18	5900 (3700, 15000)	3700 (2500, 6600)	1500 (860, 1600)	0.001*	0.02**
CSF2	30 (0, 150)	1.2 (0, 87)	4.1 (0.65, 7.1)	0.19	
IFNγ	1400 (3.4, 11000)	600 (140, 2500)	140 (0, 1100)	0.036*	0.57
TNFα	170 (50, 390)	110 (41, 150)	8.5 (2.8, 28)	0.003*	0.05**
TPO	0.065 (0, 40)	0.25 (0, 4.3	0.31 (0, 0.97)	0.68	

Pro-inflammatory cytokine levels (normalized Total Peak Area, nTPA) reported as median (min, max) for dogs with primary glaucoma (‘glaucoma group’), anterior uveitis and post-operative ocular hypertension after phacoemulsification surgery (‘POH group’), and normal eyes (‘normal group’). Reported values are nTPA E^-4^. Kruskal-Wallis test was used to compare between groups. Bonferroni’s adjustment was performed on any higher raw p-values to account for multiple variables.

*indicates significant raw p-value

**indicates significant p-value with Bonferroni’s adjustment.

## Discussion

The present study demonstrates that MRM-MS using Canine Cytokine SpikeMix™ as an internal and normalization standard can detect pro-inflammatory cytokines in canine AH. Also, IL-4, IL-6, IL-18, and TNFα may be important pro-inflammatory cytokines in dogs with anterior uveitis and POH following phacoemulsification (‘POH group’). Additionally, IL-18 and TNFα were higher in patients with glaucoma compared to the ‘POH group’. This may indicate an inflammatory component to eyes with primary glaucoma.

The current study demonstrates that IL-4, IL-6, IL-18, and TNFα have the potential to be relevant in dogs with post-phaco anterior uveitis. Dong et al. (2015) evaluated AH cytokines in human patients undergoing phacoemulsification surgery. Pre-operative samples had detectable levels of IL-4 and IL-6, while TNFα was not detectable [50]. However, Liu et al. (2019) demonstrated IL-4, IL-6, as well as IL-18 and TNFα were present in AH of human patients after phacoemulsification surgery, indicating these pro-inflammatory cytokines may be important in post-phacoemulsification anterior uveitis and POH [51]. This compliments the current study’s findings, indicating that anterior uveitis following phacoemulsification may have a similar pro-inflammatory cytokine profiles in humans and canines.

IL-18 is a proinflammatory cytokine that stimulates development of Th1 cells and increases the secretion of IFNγ [52]. Our study is consistent with the human literature regarding IL-18 involvement in anterior uveitis post-phaco [51]. This is not surprising, given that anterior uveitis is believed to be characterized by a Th1 directed immune response [21,53,54]. Conversely, IL-18 was positively correlated with IOP which could indicate that increasing IL-18 is a response to a rise in IOP rather than the anterior uveitis. TNFα, a major pro-inflammatory cytokine, was higher in canine AH of post-phaco eyes compared to the normal control eyes in the current study. In accordance with these findings, TNFα plays a major role in human uveitis and has been detected in human patients following phacoemulsification surgery [19,20,51,55,56]. Neutralization or suppression of TNFα has shown to be effective in preventing experimental autoimmune uveitis [57,58]. However, in canine patients, Pinard et al. (2011) did not find detectable levels of TNFα in AH of canines following aqueous paracentesis to induce experimental uveitis [59]. This difference may be due to differing laboratory methods, or could represent a clinical difference between canine patients with anterior uveitis and POH following phacoemulsification and experimentally induced uveitis [47].

IL-4 and IL-6 levels were higher in the ‘POH group’ compared to the ‘normal group‘ and ‘glaucoma group’, respectively. IL-4 and IL-6 have also been detected in human patients undergoing phacoemulsification [22,50,51]. Additionally, when evaluating AH from human patients with glaucoma, IL-4 has been undetectable [25,60]. Other studies in human glaucoma patients have found that IL-6 levels did not differ from the normal controls [25,61]. These findings of IL-4 and IL-6 in the human literature support that post-phaco patients with POH and glaucoma patients have a unique inflammatory response, which is likely to be applicable to our canine patients.

Treatment of anterior uveitis is often non-specific, involving topical and systemic glucocorticoids and/or NSAIDs. The use of long-term topical and systemic anti-inflammatory therapies can lead to side effects including lipid keratopathy, increased risk of ocular and systemic infections, gastrointestinal disease, renal disease, and iatrogenic hyperadrenocorticism [13,14,62]. Targeting specific pro-inflammatory cytokines has proven to be clinically beneficial in several autoimmune disorders in human medicine [63]. TNFα is a major target for treating both ophthalmic and systemic inflammatory and autoimmune disease [64,65]. HUMIRA™, a human monoclonal antibody ‘adalimumab’ is a TNFα inhibitor, which has shown positive results in the treatment of uveitis in humans [66]. In rats, CBD (Cannabidiol) has demonstrated inhibition of TNFα in experimentally induced uveitis as well as retinal TNFα release, which could be efficacious in management of autoimmune uveitis, retinal inflammation, and neuroprotection [67]. Other immunotherapies to treat human uveitis are being investigated, and newer studies with Janus kinase (JAK) inhibitors have shown promising results [17]. JAK inhibitors block multiple pro-inflammatory cytokines implicated in human uveitis such as IL-6 and IFNγ, resulting in disruption of Th1 differentiation [64,68,69]. These new therapies may be useful in our canine patients and further studies investigating effectiveness are warranted. Additionally, the use of target mass spectrometry could be utilized to detect inflammatory cytokines in treated versus untreated eyes, serving as an outcome measure for these future investigations.

Interestingly, AH levels of IL-18 and TNFα were higher in glaucoma eyes than POH eyes following phacoemulsification surgery. Patients with primary glaucoma were chosen as an IOP-matched group to evaluate for pro-inflammatory cytokines related to IOP rise rather than anterior uveitis and POH. There was no significant difference in IOP between the ‘POH group’ and ‘glaucoma group’ at the time of AH collection. Many studies show minimal to no detection of TNFα in AH of human glaucomatous eyes, but it has been suggested that TNFα levels may be higher in the vitreous humor compared to the AH [70]. Sawada et al. (2009) suggests that TNFα may signal neuronal damage in glaucomatous eyes due to secretion of TNFα from stressed posterior segment cells such as astrocytes, microglial cells, and retinal Müller glial cells [71–74]. Glaucoma patients in our study had end-stage, blind eyes with severe retinal atrophy and optic nerve head cupping on histopathology, possibly resulting in higher TNFα levels than reported in humans. Although the IOP did not differ between the ‘glaucoma group’ and ‘POH group’ at the time of AH sampling, when evaluating the highest reported IOP values from each patient, the ‘glaucoma group’ had significantly higher IOP values than the ‘POH group’. Additionally, IL-18 levels were positively correlated with increasing IOP. This may account for the higher pro-inflammatory cytokine values in the the ‘glaucoma group’ compared to the ‘POH group’, suggesting that IL-18 may be associated with increased IOP rather than anterior uveitis and POH. Further investigation is needed to determine the pathophysiology of increased TNFα and IL-18 in primary glaucoma eyes.

In a study evaluating the histopathology of canine eyes with goniodysgenesis, over 85% of patients with acute glaucoma were found to have inflammatory cells in the trabecular meshwork. Therefore, the authors suggest that acute inflammation may play a pivotal role in the pathogenesis of the disease [75]. A more recent study evaluating the efficacy of anti-inflammatory therapy in patients with goniodysgenesis found an increased eye survival time in patients treated with anti-glaucoma and anti-inflammatory therapies, versus anti-glaucoma therapy alone [76]. However, these results were not statistically significant. The presence of major pro-inflammatory cytokines in the AH of canines with chronic primary glaucoma, may indicate anti-inflammatory therapies could be a beneficial adjunctive therapy to anti-glaucoma medications. However, this information should be interpreted with caution, as the small sample size does not allow validation of these conclusions, only chronic glaucomatous eyes were evaluated, and topical steroids have been associated with increased IOP in dogs [77].

Treatment for primary glaucoma involves various medical or surgical therapies aimed at delaying elevations in IOP and preserving vision for as long as possible [78]. However, elevations in IOP are inevitable and will eventually result in blind and painful eyes [78]. Therapeutics for primary glaucoma are an active area of research in both human and veterinary medicine [78,79]. A recent study by Plummer et al. (2021) demonstrated that 37.7% of clinicians will treat primary glaucoma patients prophylactically with a topical anti-inflammatory [80]. However, the role of inflammation in primary glaucoma is largely unknown. A better understanding of pro-inflammatory cytokines involved in primary glaucoma may lead to advancements in medical therapy.

Most patients in our study were on topical or systemic anti-inflammatories prior to collection of AH which is likely to alter the cytokine profile. However, this data remains clinically relevant as treatment with topical anti-inflammatories post-phacoemulsification is gold standard. Analysis of the cytokines that remain elevated despite anti-inflammatory treatment may help direct future therapies to better control anterior uveitis and POH following phacoemulsification. Additionally, post-phaco and glaucoma patients were prescribed medications based on the individual patient needs and were not standardized in this study. Other medications, such as topical anti-glaucoma medications or antibiotics, may change the AH cytokine profile in canine patients, altering the results of the data in the present study. Latanoprost was given in a large number of patients prior to AH collection. Half the patients in the ‘glaucoma group’ (3 out of 6 eyes) were receiving topical latanoprost twice daily and 8 out of 10 eyes in the ‘POH group’ were receiving latanoprost as first line treatment for POH (Table 2). Latanoprost, a prostaglandin analog, decreases IOP by increasing AH outflow via the uveoscleral pathway but also results in breakdown of the BAB [81,82]. This could have resulted in elevated levels of cytokines in AH of both our glaucoma and POH patients.

Steroid-induced ocular hypertension is well documented in human patients [83]. Additionally, there is no significant difference in post-phacoemulsification anterior uveitis in humans when treated with topical NSAIDs versus corticosteroids [84]. However, the use of topical corticosteroids following phacoemulsification is common in veterinary ophthalmology to limit surgical-induced anterior uveitis and prevent the development of secondary glaucoma [6,43,44,85–87]. In canine patients, oral hydrocortisone and intravitreal triamcinolone do not induce elevations in IOP [88,89]. One canine study has demonstrated ocular hypertension in glaucomatous beagles with the use of topical dexamethasone [77]. Although we cannot rule out corticosteroids as a risk factor for POH development, control of post-operative uveitis is essential for a successful outcome following phacoemulsification in canines [87].

Retention of viscoelastic material following cataract surgery is a risk factor for the development of POH and is difficult to differentiate from POH induced by anterior uveitis [11]. Although, a study by Klein et al (2011), demonstrated a significant reduction in the incidence of POH when irrigation and aspiration of viscoelastic material was performed for 1 min at the end of phacoemulsification surgery [6]. In our study, irrigation and aspiration was performed for 1 min in all eyes to reduce the incidence of POH caused by retained viscoelastic material. Additionally, only eyes with signs of anterior uveitis were included. However, ensuring 100% removal of viscoelastic is not possible, making this a limitation of the study.

One inherent limitation of this study was the small sample size, making our results preliminary. A larger number of AH samples may lead to more statistically significant differences between groups. Additionally, we were unable to evaluate any potential differences between hereditary and diabetic cataracts following phacoemulsification surgery. A future study with more cases could evaluate pro-inflammatory cytokines in relation to blood glucose levels and serum pro-inflammatory cytokines collected at the same time as AH samples. Although many studies show no difference in post-operative outcomes between diabetic and non-diabetic patients, their cytokine profiles may differ [85,86,90]. This study involved simultaneous analysis of multiple factors (cytokines) from each sample. The Bonferroni correction was applied in this study to determine the significance of changes in pro-inflammatory cytokine levels. This statistical adjustment is conservative, and it may mask potential changes in the data.

Another limitation of our study is that phacoemulsification surgery was performed by either a DACVO or an ophthalmology resident under direct supervision of a DACVO. It is possible that increased surgery time could result in higher post-operative inflammation and complications in eyes with an ophthalmology resident performing surgery. However studies in dogs have demonstrated that higher phacoemulsification times do not correlate an increased risk of complications, such as glaucoma and POH [6,43]. Correlation between surgeon experience and the proinflammatory cytokines in canine aqueous humor was not evaluated and requires future research with a larger sample size. Additionally, even experienced surgeons can come into complications and longer surgery times making these results clinically relevant. Lastly, one dog in our study with goniodysgenesis on gonioscopy and bilateral blindness did not receive necropsy exam and histopathology of the globes. Although, gonioscopy is a primary diagnostic method in the evaluation of human and veterinary patients with glaucoma, it does not allow evaluation of the deeper filtration angle structures [91]. The lack of histopathology on this patient is unlikely to affect our results given there were no outlier pro-inflammatory cytokine values in the ‘glaucoma group’.

In conclusion, MRM-MS using the Canine Cytokine SpikeMix™ was able to detect levels of pro-inflammatory cytokines in canine AH. Dogs with anterior uveitis and POH following phacoemulsification surgery express elevated levels of IL-4, IL-6, IL-18 and TNFα in AH, which is consistent with the human literature. Additionally, dogs with glaucoma had higher levels of IL-18 and TNFα in AH compared to dogs with anterior uveitis and POH following phacoemulsification. This may indicate inflammation plays a role in the pathogenesis of glaucoma. The findings in this study emphasize the differential inflammatory responses and pro-inflammatory cytokine footprints in patients with glaucoma and POH following phacoemulsification.

## Supporting information

S1 Table Pro-inflammatory cytokine levels. Pro-inflammatory cytokine levels (normalized Total Peak Area, nTPA) for the ‘glaucoma group,’ ‘POH group,’ and ‘normal group.’ This table displays the sample identification, dog number (corresponding to Table 1), study group, IOP at the time of aqueous humor collection, and nTPA for all cytokines.
(XLSX)

S2 Table Statistical analysis of pro-inflammatory cytokine levels. The “SumStats” table displays the summary statistics for each cytokine (normalized Total Peak Area, nTPA) and group including median, minimun, maximum, and n = the number of samples in each group. The “Q1Result” show the comparison between all groups for each cytokine including the raw *p*-value (KW_Raw_p) and Bonferroni adjusted *p-*value (KW_Bon_P). It also includes raw *p-*values for pairwise comparisons between groups (DSCF_Raw_P). The “Q2Results” shows the correlation between IOP and each cytokine including n = number of samples in correlation, Spearman correlation (SpearCorr), raw *p-*value (Spear_Raw_p), and Bonferroni adjusted *p-*value (Spear_Bon_p).
(XLSX)
